# Cardiac Tamponade in Concurrent Sickle Cell Disease and Systemic Lupus Erythematosus: An Unusual Association

**DOI:** 10.7759/cureus.55285

**Published:** 2024-02-29

**Authors:** Jude Elsaygh, Marie Liu, Akhaled Zaher, Andrew Treihaft, Laura Bradel

**Affiliations:** 1 Internal Medicine, NewYork-Presbyterian Brooklyn Methodist Hospital, New York, USA; 2 Cardiology, NewYork-Presbyterian Brooklyn Methodist Hospital, New York, USA

**Keywords:** sickle cell disease (scd), pericardial effusion, cardiac tamponade, systemic lupus erythema, lupus

## Abstract

This case report describes a rare occurrence of the coexistence of sickle cell disease (SCD) and systemic lupus erythematosus (SLE) in a 33-year-old female. The overlapping clinical manifestations posed diagnostic challenges, leading to a delayed diagnosis. The patient's presentation with pericardial effusion and tamponade during a concurrent SLE flare highlights the complexity of managing these conditions. The case underscores the importance of heightened clinical awareness and multidisciplinary collaboration for accurate diagnosis and timely intervention in such rare comorbidities.

## Introduction

The coexistence of sickle cell disease (SCD) and systemic lupus erythematosus (SLE) is of interest but seems to be a rare association, as only approximately over 40 similar cases have been reported in the literature in the last 50 years [1]. Individuals of African, Afro-Caribbean, or African-American descent, particularly women, are at a higher risk of developing both SCD and SLE as independent diseases. A previous study examining the overlap of these conditions within the same individual found that 73% of affected individuals were Black women [2]. Reported cases have shown patients to have been diagnosed with SCD for several years before SLE, with articular involvement as the most frequent lupus-related symptom (85%), followed by serositis (36%) and glomerulonephritis class III or IV (11%) [1]. 

Individuals with SCD are known to have a higher likelihood of contracting infections. Deficiencies of the complement system were first studied by Johnston et al. to understand this increased risk of infection. Through prevention of the activation of C1 and the classic complement sequence, they observed that individuals with SCD did not fully activate and attach the opsonin C3 to foreign microorganisms via the alternate complement pathway [3].  Individuals with SCD were determined to have dysfunctional activation of the alternate pathway of the complement system, which heightens their susceptibility to infections caused by encapsulated bacteria and impedes their ability to clear antigens. Some authors have posited that this increases the susceptibility of those with SCD to developing autoimmune disorders [4,5]. This makes the diagnosis of the etiology of pericardial effusion in this population subset complex and interesting to learn about. 

Cardiac tamponade, a sequela of pericardial effusion, is particularly challenging to diagnose in patients with concurrent SLE and SCD. Patients with SCD often complain of shortness of breath, especially during times of sickle cell crisis. This complaint often alerts physicians to acute chest syndrome, but it is important to evaluate for cardiac tamponade to avoid missing this life-threatening diagnosis. This case will delve deeper into the clinical course of a young female with concurrent SCD and SLE who developed cardiac tamponade. 

## Case presentation

**Figure 1:**
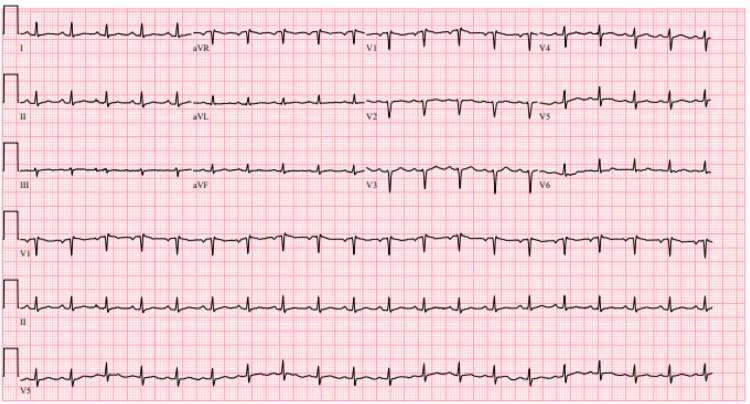
The EKG shows sinus tachycardia with low voltage and electrical alternans.

**Figure 2:**
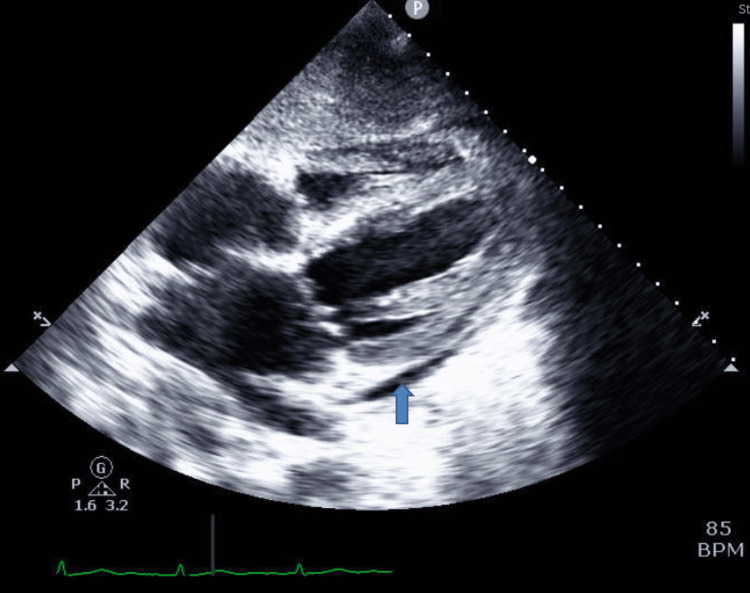
The initial transthoracic echocardiogram on admission shows a small pericardial effusion (blue arrow) on the parasternal long-axis view.

A 33-year-old African American female presented with progressive chest pain, polyarthralgia, and limb swelling for three days. A physical exam was significant for a temperature of 39.4°C, tachycardia to a heart rate of 119 beats per minute (bpm), systolic murmur, bibasilar rales, bilateral shoulder and hip tenderness, and polyarticular effusions. Her medical history included SCD and SLE on hydroxychloroquine and mycophenolate. The EKG showed sinus tachycardia with low-voltage QRS and no evidence of ST/T wave changes or PR depression (Figure 1). A transthoracic echocardiogram performed at the time of admission revealed a small pericardial effusion (Figure 2). Laboratory results showed mild leukocytosis at 11.5 x 10^3^/ μL and normal procalcitonin and troponin levels. A chest X-ray revealed a left pleural effusion and patchy consolidation concerning multifocal pneumonia. The CT-pulmonary embolism protocol revealed patchy consolidation, ground-glass opacities within the lingula, a small left effusion, and no evidence of pulmonary artery dilatation or pulmonary embolism. Sepsis protocol was initiated, and she was started on empiric antibiotics, steroids, and a pain regimen for concern of pneumonia triggering a sickle cell crisis and SLE flare. 

**Figure 3:**
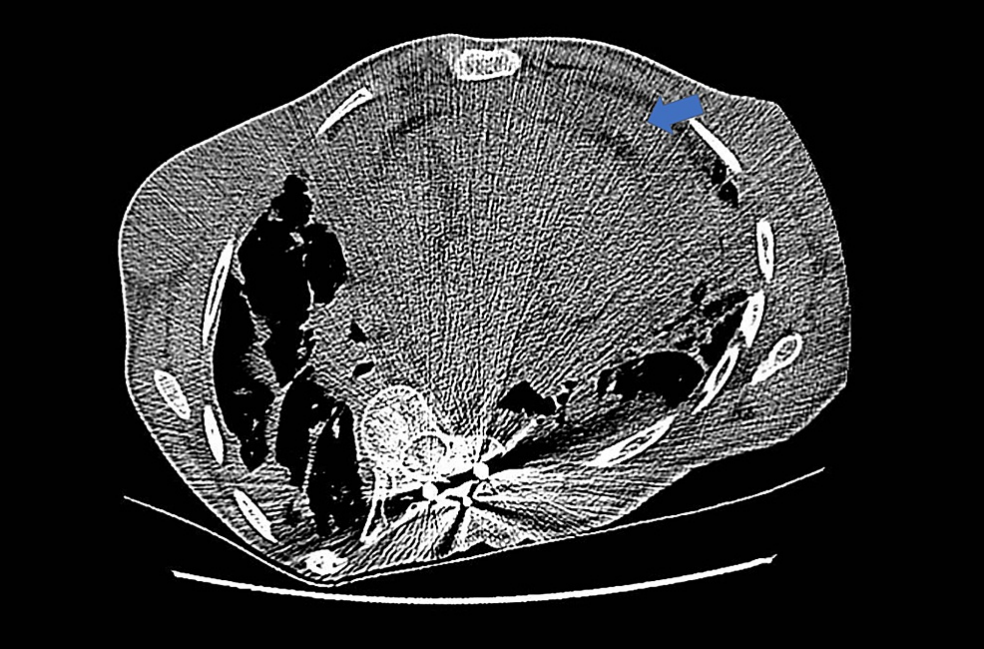
The chest CT shows bibasilar consolidative opacities indicating an infection. A new moderate pericardial effusion is seen (blue arrow).

**Figure 4:**
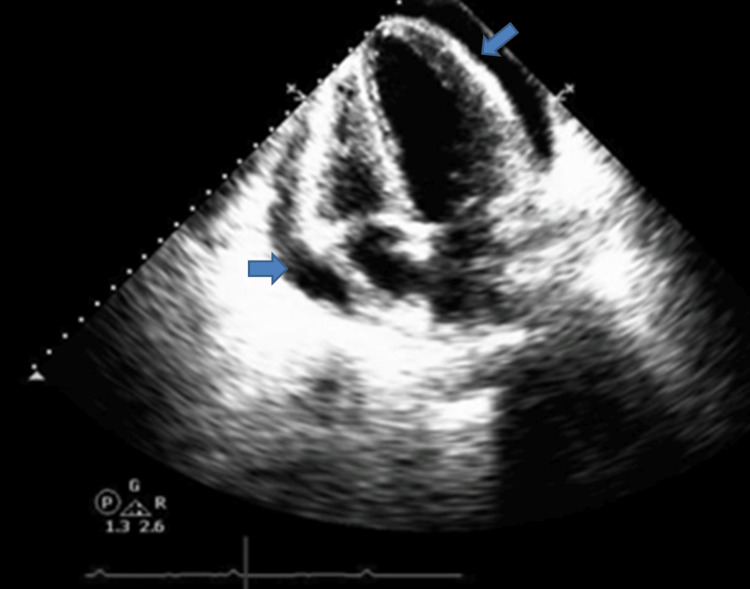
A transthoracic echocardiogram in the apical four-chamber view shows rapid enlargement of a large circumferential pericardial effusion (blue arrow) with right atrial free wall buckling and right ventricular systolic obliteration.

**Figure 5:**
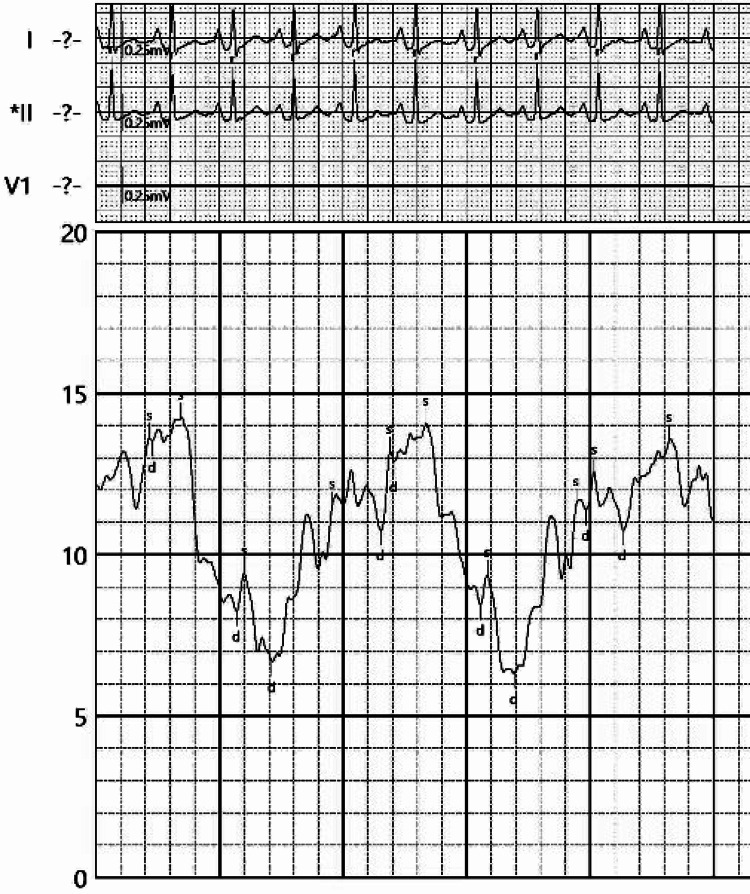
Elevated pericardial pressure at 10 mmHg prior to pericardiocentesis

**Figure 6:**
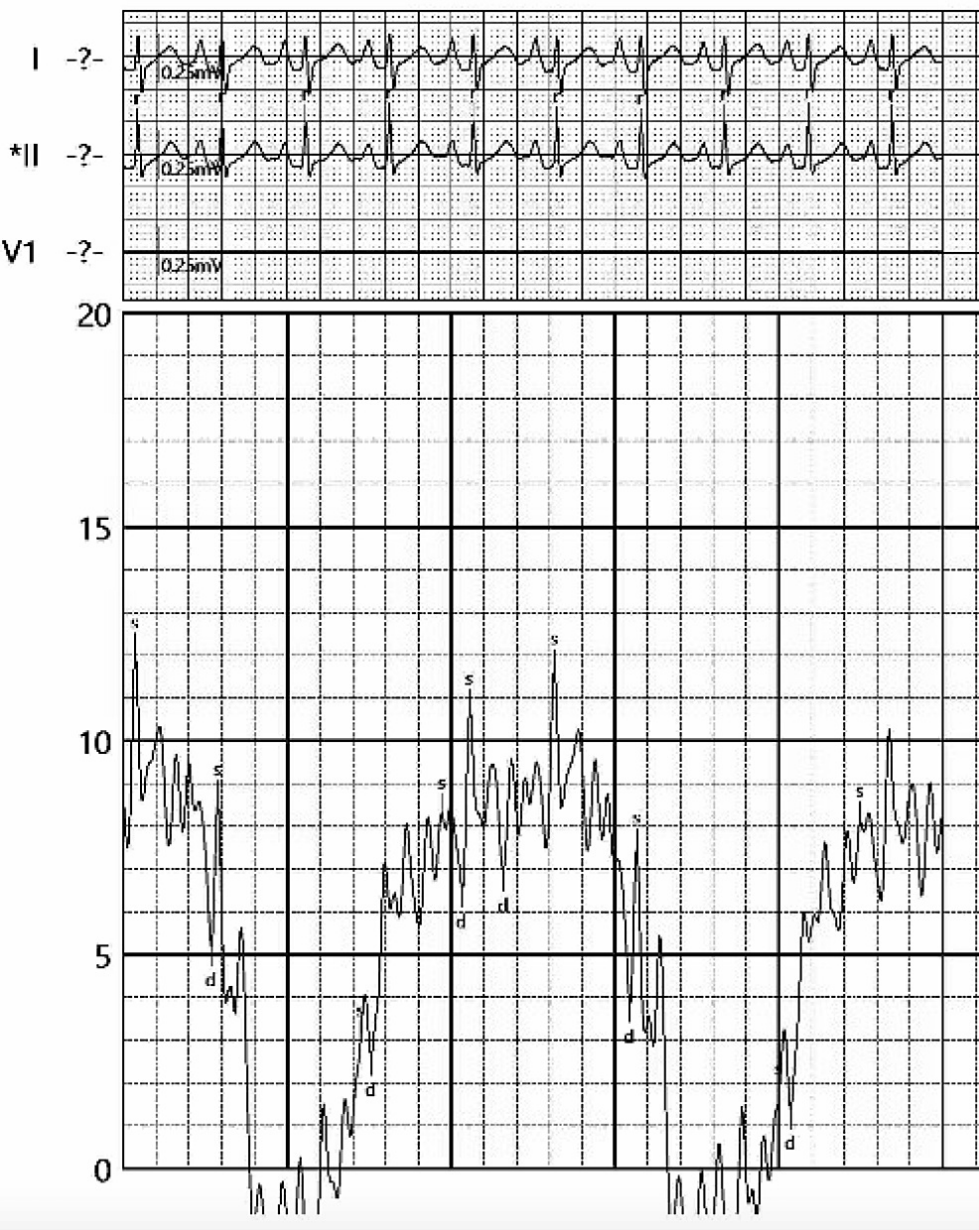
A decrease in pericardial pressure to 4 mmHg post pericardiocentesis

**Figure 7:**
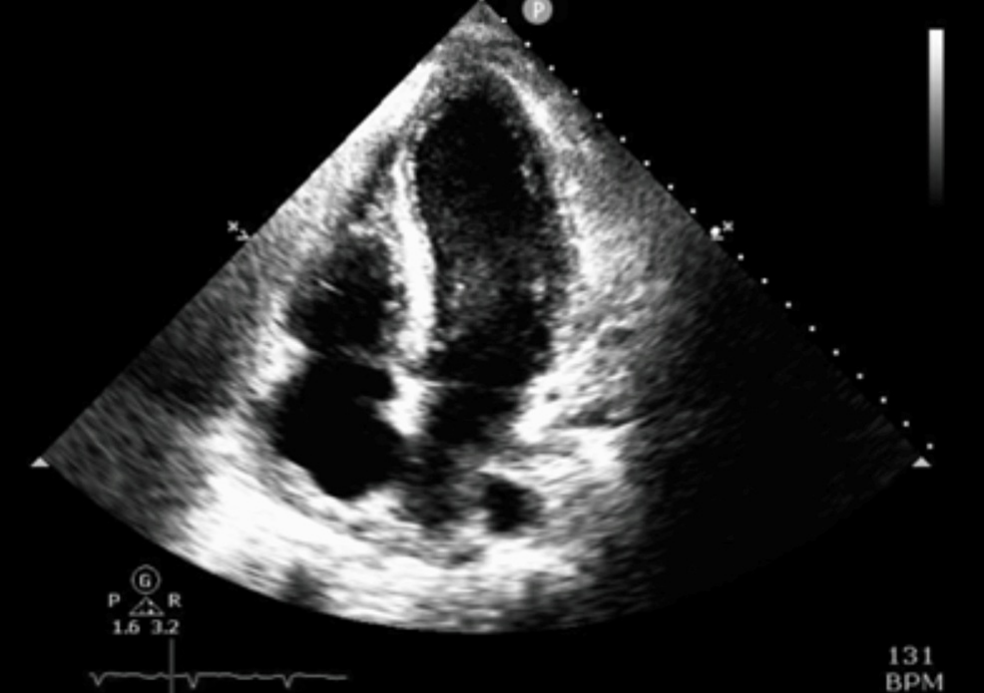
The apical four-chamber view shows complete resolution of pericardial effusion.

Her hospital course was complicated by recurrent fevers, tachycardia, dyspnea, and hypoxia with worsened chest pain. A repeat chest X-ray revealed worsening consolidations; a CT of the chest showed a moderate pericardial effusion (Figure 3). A repeat echocardiogram revealed a large pericardial effusion with tamponade physiology showing right atrial free wall buckling, right ventricular systolic obliteration, and inspiratory variations (Figure 4). Cardiology was consulted, and she underwent emergent pericardiocentesis using a subxiphoid approach with fluoroscopy. A decrease in mean pericardial pressure from 10 mmHg to 4 mmHg post pericardiocentesis is shown in Figures 5-6. The patient had marked improvement in her shortness of breath after the pericardiocentesis. Pericardial fluid studies appeared inflammatory in etiology, more likely from SLE flare than infectious etiology. After a pulse dose of steroids at 10 mg/kg and a new rituximab infusion, her dyspnea and chest pain symptoms improved. Her transthoracic echocardiogram at the time of discharge showed resolution of the pericardial effusion (Figure 7). She was discharged home on a steroid taper with outpatient rituximab infusions and close follow-up with cardiology, rheumatology, and her primary care physician.

## Discussion

Initially, there was no strong association in the literature linking SCD and SLE, although they have several overlapping clinical manifestations, including arthritis, anemia, fever, and renal, cardiovascular, and pulmonary involvement. However, there is increasing co-existence between SCD and SLE, which is not so rare anymore. The incidence of connective tissue diseases such as SLE in adult patients with SCD appears to be increasing. The exact causes underlying this increased risk are still unknown, but a link with B regulatory (Breg) cells is possible as these cells suppress inflammatory responses and maintain tolerance [6]. Few published case reports have linked pericardial effusion as a rare complication in SCD [7, 8]. Diagnosing concurrent SCD and SLE is a challenge, as approximately 20% of SCD patients demonstrate positive antinuclear antibodies (ANAs) with titers greater than 1/160 [9]. An observational cohort study in London suggests a significantly increased incidence of connective tissue disorders like SLE in patients with SCD compared to the general population of a similar ethnic background [10]. However, the exact reasons for this heightened risk remain unclear. 

Cardiac tamponade has not been described in the literature for SCD patients. The majority of cardiac manifestations of SCD involve pulmonary hypertension and diastolic heart failure [11]. The diagnostic challenge in identifying cardiac tamponade in this patient population is that acute chest can present similarly to cardiac tamponade, with chest pain and shortness of breath [12]. This is primarily the reason we decided to write this case report: to inform clinicians of this possible association. Earlier recognition of this life-threatening clinical condition could be lifesaving. 

Cardiac tamponade is a life-threatening condition where pericardial effusion causes an impairment of the diastolic filling of the ventricles, therefore, compromising cardiac output. The characteristics of Beck’s triad for its recognition on a physical exam are hypotension, elevated jugular venous pressure, and muffled heart sounds. In the era of point-of-care ultrasounds, a large pericardial effusion can be assessed in a time-efficient manner. A combination of either atrial or ventricular collapse can be seen in up to 90% of cases of cardiac tamponade [13]. The recognition of cardiac tamponade prompts an urgent pericardiocentesis to relieve the pressure within the pericardiac sac, often accompanied by a pericardial drain. Further laboratory testing of the pericardial effusion specimen can clue the physician to the etiology, in our case, showing an inflammatory pattern consistent with an SLE flare. A repeat transthoracic echocardiogram is useful in assessing for re-accumulation of fluid and is used to time the removal of the pericardial drain. 

## Conclusions

Further investigation into the possible association between SLE and other connective tissue diseases and SCD is warranted for greater diagnostic vigilance among providers. It is imperative that clinicians promptly recognize and treat life-threatening complications associated with each disease when they appear concurrently, as they might require more aggressive management.
